# Pitfalls of hip hop pedagogy: Re-examining and questioning the definition

**DOI:** 10.3389/fpsyg.2023.1135808

**Published:** 2023-03-28

**Authors:** Ming Tao Wei, Zhi Yang, Xin Wang, Dan Xi Liu, Yu Jie Bai, Rui Yi Guo, Ning Yu, Xi Ling

**Affiliations:** ^1^Teachers College, Changshu Institute of Technology, Suzhou, China; ^2^Melbourne Graduate School of Education, The University of Melbourne, Melbourne, VIC, Australia; ^3^ShaanXi Branch of Guangzhou Bosha Architectural Design Institute, Xi’an, China; ^4^Faculty of Fine-Applied Arts and Cultural Science, Mahasarakham University, Maha Sarakham, Thailand; ^5^Faculty of Education, Shaanxi Normal University, Xi’an, China; ^6^Department of Physical Education, Fuzhou Institute of Technology, Fuzhou, China

**Keywords:** hip-hop pedagogy, assessment, formal learning environment, education, rubric/graded scoring key, pedagogy, hip-hop based education

## Abstract

Recently, hip-hop pedagogy or Hip-Hop Based Education (HHBE) have become buzz words in the academic and public debate around hip-hop. However, we found that most definitions of hip-hop pedagogy are missing the concept of pedagogy itself. One consequence of failing to adequately explain the concept of pedagogy is that it may lead future hip-hop researchers, students, and teachers inadvertently to disseminate misinformation or foster unclear thinking by using “hip-hop pedagogy” in inaccurate or vague ways. For these reasons, it is important to have a shared understanding of hip-hop pedagogy. In this article, we present three updated, expanded definitions of hip-hop pedagogy with the potential for widespread acceptance. These definitions aim to convey in the simplest terms what hip-hop pedagogy is for the purpose of informing educators and preparing them to use data.

## Introduction

“If names are not right then speech does not accord with things; if speech is not in accord with things, then affairs cannot be successful.” (Confucius, The Analects)

In recent years, research into the use of hip-hop pedagogy to increase student’s engagement in class and interest in course material has been of increasing interest. Many studies have demonstrated that hip-hop pedagogy stimulates students’ critical thinking (Kelly, 2013; Villanueva, 2022), gives students a better understanding of what they learned from a lecture (Adjapong and Emdin, 2015; Adjapong, 2021; Hains et al., 2021), and provides a sense of accomplishment (Hall and Martin, 2013; Richards, 2021; Jones, 2022). Hip-hop pedagogy scholar from Columbia University, Adjapong, and his colleagues defined hip-hop pedagogy as the following:

Hip-Hop pedagogy as a way of authentically and practically incorporating the creative elements [graffiti art, MCing, breakdance, DJing, and knowledge] of Hip-Hop into teaching, and inviting students to have a connection with the content …, and through their realities and experiences. (Adjapong and Emdin, 2015, p. 67)

In their study, they adopted focus groups and open-ended questionnaires to collect the attitudes toward hip-hop pedagogy from approximately 35 students in science classrooms and discussed with students how hip-hop pedagogy supported them in learning science content (Adjapong and Emdin, 2015). This research led the authors to conclude that hip-hop pedagogy was well received by the students and deepened “their understanding of the scientific content” (Adjapong and Emdin, 2015, p. 73).

Notably, although the study by Adjapong and Emdin (2015) did confirm that hip-hop pedagogy was well received by the participants, the available data were not sufficient to draw firm conclusions about hip-hop pedagogy increasing student understanding of science content. Because their study did not describe details about the syllabus and provide judgment criteria about whether students truly understood the science content (Adjapong and Emdin, 2015). In this case, we believe that challenging questions need to be asked to minimize the risks of invalidating conclusions stemming from insufficient data. These questions include how teachers or researchers know if students’ responses are correct (or false), does the article provide explicit teaching guidelines and judging criteria, what are these criteria, are these criteria delivered to students before the course starts (or at the end of the course), how do teachers know if students are cheating, to what extent is students’ understanding of the content of scientific knowledge related to the hip-hop pedagogy?

In addition to the problem of insufficient data, we also found that Adjapong and Emdin’s (2015) definition of the term hip-hop pedagogy was both ill-conceived and rested on shaky foundations. One major reason is that their definition of hip-hop pedagogy missing the concept of pedagogy itself. For instance, their definition of hip-hop pedagogy focused on “the creative elements of Hip-Hop,” the students’ experiences and culture, and the content of the teaching (Adjapong and Emdin, 2015, p. 67), but did not even include the learning purposes and results frequently mentioned in definitions of pedagogy (Best, 1988; Hinchliffe, 2000; Alexander, 2008; Shah and Campus, 2021). One question needs to be asked again here. Where is the pedagogy in the definition of hip-hop pedagogy?

Furthermore, the problem of unexplained use of the terms pedagogy and education appears several times in Adjapong and Emdin (2015)’s article, such as “Hip-Hop Based Education” (pp. 66, 67), “hip hop pedagogy” (pp. 66, 67, 68, 70, 72, 76), “the pedagogy of Hip-Hop” (p. 67), “incorporating Hip-Hop in education” (p. 69), “incorporating Hip-Hop pedagogy in the science classroom” (p. 75) and “Hip-Hop as a teaching approach” (p. 67). While all these words mean “to combine into a more or less uniform whole,” mix may or may not imply the loss of each element’s identity. Again, the mix and match among these terms beg several conceptual questions. What is the pedagogy? What is the difference between pedagogy and education? Is it appropriate to match the terms hip hop and pedagogy? If so, what makes a good rationale? If not, does this match imply a loss of identity for each element (hip-hop or pedagogy)?

Regrettably, the article by Adjapong and Emdin (2015) does not clarify the meaning between pedagogy and education nor provide a rationale for combining the terms hip-hop and pedagogy. One consequence of failing to explain the meaning of pedagogy adequately is that this phenomenon may lead future hip-hop researchers, students, and teachers inadvertently to disseminate misinformation or foster unclear thinking by using “hip-hop pedagogy” in inaccurate or vague ways.

In this context, we want to activate a lively discussion about the definition of hip-hop pedagogy and revise the existing hip-hop pedagogy definitions by recovering and restating those ideals associated with pedagogy. We argue that the definition of hip-hop pedagogy should include clear domain delineation of the concept to avoid overlapping and confusing terms. It must include an explanation of the term pedagogy and should clearly articulate whether the whole point of learning is to equip people for specific social or economic requirements.

## Methods

With interest in ways we can do better, we focus neither on demonstrating the volume of the literature nor on illustrating the perceptions about hip-hop history contained within particular branches of this literature. Rather, our goal is to approach the literature from a new direction: (a) determining the definition of pedagogy, (b) identifying the different ways the term hip-hop is used to define the concept of hip-hop pedagogy, and (c) proposing alternative narratives designed to promote a better understanding of the concept of hip-hop pedagogy. To accomplish these goals, we draw on scholarship related to pedagogy to understand hip-hop pedagogy (Best, 1988; Hinchliffe, 2000; Canning, 2007; Alexander, 2008).

In this article, we start by collecting definitions of pedagogy to develop a common understanding of what pedagogy is and then analyze the usage of the term hip-hop in the context of hip-hop pedagogy to understand the definition of hip-hop pedagogy better. Finally, we present an updated and expanded template for the definition of hip-hop pedagogy to convey in the simplest terms what hip-hop pedagogy is for the purpose of informing educators and preparing them to use data.

### Definitions and descriptions of pedagogy

Some papers indicated an intention to focus on pedagogy *via* their titles, keywords, or abstracts. For example, pedagogy can be explained as a discipline with academic status (Marton, 1997; Canning, 2007; Stephens, 2009), teaching action (Nolinske and Millis, 1999; Moate and Cox, 2015), and linked to economic and social development (Best, 1988; Hinchliffe, 2000). Regardless of whether these papers focused on multiple or single definitions, each paper included in the review reflected the following claims about the definition of pedagogy:

1. Pedagogy can be defined as learning oriented toward social needs. Learning aims to prepare students for the talent market and help them develop job-related skills while growing personally and intellectually.
2. As pedagogy, the results of learning must be measurable because the whole meaning of learning is to enable people to meet specific social, political, and economic requirements.

We illustrate each of these claims here by referencing a range of papers from different contexts and focusing on different interpretations.

With respect to the first knowledge claim, Shah and Campus (2021) discusses the meaning of pedagogy from etymological aspects. For example, Shah and Campus (2021) noted:

The word pedagogy has its roots in Ancient Greece. Rich families in Ancient Greece would have many servants, often slaves, one of whom would be specifically tasked to look after the children. Often these slaves would lead or escort the children to the place of education. (p. 26)

According to Shah and Campus (2021), the term “pedagogy” seems connected with ideas of supervision, discipline, and conformism to make students useful members of society (Knowledge Claim 1). From the etymological perspective, teachers will closely supervise students’ learning behaviors and outcomes. Alexander (2008) explains the purpose and definition of pedagogy by analyzing the etymological features of pedagogy. For instance, Alexander (2008) wrote:

Pedagogy is not a mere matter of teaching technique. It is a purposive cultural intervention in individual human development which is deeply saturated with the values and history of the society and community in which it is located. (p. 92)

Alexander’s point is that the understanding of pedagogy should not be limited to teaching approaches; it is concerned with how to help students adapt to society by purposefully organizing learning activities that are culturally relevant to their culture. In fact, Alexander’s view also responds to the fact that pedagogy can be explained as learning oriented to social needs.

Hinchliffe (2000) further explains the relationship between learning activities and social evolution processes from the perspective of instrumental learning. For example, Hinchliffe (2000) noted that “[From instrumental learning perspective], pedagogy places learning at the service of government, political power and the economy” (p. 31). Here, Hinchliffe (2000) suggests that pedagogy should focus on how learning activities can equip people with the skills needed by their country’s businesses, government, and society. This perspective also echoes the commitment that “pedagogy can be defined as learning oriented toward social goals” (Hinchliffe, 2000, p. 31) (Knowledge Claim 1). Having thus defined pedagogy, he went on to discuss the difference between “pedagogy” and “education” by reviewing the etymological features of pedagogy, the ideas of Aristotle and Dewey. For example, Hinchliffe (2000) claims that:

Construed as education, the results of learning can never be measured according to a common standard. But construed as a pedagogy, those results must be measurable because the whole point of learning is to equip people for specified social, political, and economic requirements. (p. 31)

Here, Hinchliffe (2000)’s point is that there is a distinction between education and pedagogy, and it is important to recognize that the learning outcomes of pedagogy must be measurable. That is, if researchers match the terms “hip-hop” and “pedagogy” to form “hip-hop pedagogy,” they should consider pedagogy’s feature that learning results must be measurable (Knowledge Claim 2). To this end, in hip-hop pedagogy, teachers must have clear and observable indicative behavioral criteria that validly measure the desired behavior or outcomes and associated target. So, what do assessment criteria look like? How can instructors use the assessment criteria to measure students’ academic achievement? In a study of the relationship between hip-hop pedagogy and student engagement, Hall and Martin (2013) demonstrated the role of assessment criteria in assisting teachers in observing student behavioral features. Similarly, in early 2022, we published an article in *Frontiers in Psychology* entitled Shaping the Future Direction of Breakdancing Teaching (Wei et al., 2022). This article described the entire process of designing assessment criteria (Wei et al., 2022). Given space limitations, an in-depth discussion of these findings is impractical. We invite interested readers to read it.

Then, to sum up, the first point we make about the literature is that the term “pedagogy” seems to be connected with ideas of supervision, discipline, and conformism to make students useful members of society. From the etymological perspective, teachers will closely supervise students’ learning behaviors and results. Here, it is necessary to realize that if student behaviors are directed toward achieving success under the teachers’ supervision, then the teachers’ efforts to create a climate of self-directed learning and individual responsibility will be frustrated. Secondly, from an instrumental learning perspective, the purpose of learning is to meet the requirements of specific societies and economies. In this context, we define pedagogy as learning oriented to social needs. Furthermore, we believe that if the terms “hip-hop” and “pedagogy” are matched to form a new term “hip-hop pedagogy,” then the etymological features of pedagogy should be considered. That is, the results of learning must be measurable.

Of course, we hold out little hope for a grand consensus in academia because there are so many conceptual definitions and disagreements about what pedagogy “is.” However, theorizing could be made tighter, less confusing, and more consistent if authors would talk in terms of the more specific lower-level constructs that often appear in pedagogy definitions. Next, we will discuss the usage of the key term hip-hop in the context of hip-hop pedagogy.

### Comments on the use of the term hip-hop

As mentioned in the methods section, to accurately explicate the concept of hip-hop pedagogy, in addition to considering the concept of pedagogy itself, we also need to consider the concept of hip-hop itself. In fact, we share with authors such as Hill (2009) and Kruse (2014) the firm belief that regardless of what term researchers use when talking about hip-hop pedagogy, hip-hop is a crucial part of the definition; It is considered an effective medium for stimulating interest in learning or to enhance interaction (Kruse, 2014; Buffington and Day, 2018; Adjapong, 2021; Wheatley, 2022).

According to most, but not all, studies of hip-hop pedagogy (Kruse, 2014; Buffington and Day, 2018; Adjapong, 2021; Brown, 2021; Wheatley, 2022), we found that the key element hip-hop is used to define hip-hop pedagogy (Anyiwo et al., 2022; Griffith et al., 2022; Guanci, 2022; Uca et al., 2022) in the following ways:

1. Learning organized or delivered through hip-hop elements
2. Incorporate knowledge related to hip-hop into teaching materials
3. Use of cipher to enhance interaction

We illustrate here the usage of the key element hip-hop in the context of hip-hop pedagogy by referencing a series of papers taken from different contexts.

We begin with the first of these points. In the definition of hip-hop pedagogy by Adjapong (2021), the five elements of hip-hop, such as graffiti, break dancing, DJing, MCing, and self-knowledge, have become key components in defining the concept of hip-hop pedagogy. Taking graffiti art as an example. Adjapong (2021) noted that “When utilizing hip-hop pedagogy, students are charged with tasks where they engaged in the visual arts, similar to graffiti artists, to work through and demonstrate their understanding of science content” (p. 850). Here, graffiti, as a hip-hop element, is used to convey students’ thoughts and emotions.

This pattern is also illustrated in the article by Wheatley (2022). Focusing on the “core tenets of hip-hop pedagogy” and located in the United States (Wheatley, 2022, p. 104). Wheatley (2022) begins by acknowledging hip-hop as a key element in defining hip-hop pedagogy. For example, Wheatley (2022) writes:

Hip-hop pedagogy is an approach to teaching that synthesizes hip-hop music, hip-hop culture, and student-centered teaching to foster student empowerment and creativity, and it is an effective approach to supporting and celebrating Black queer students. (p. 104)

Notice here that the key element hip-hop is described as hip-hop music and hip-hop culture. This finding is significant because it suggests that knowledge related to hip-hop, such as hip-hop music and hip-hop culture, should be included in the definition of hip-hop pedagogy. In fact, we share with the authors such as Adjapong (2021) and Wheatley (2022) the firm belief that if hip-hop elements are applied appropriately, they can play a powerful role in teaching students better.

The article by Buffington and Day (2018) provides a third example for understanding the usage of the key element hip-hop in defining hip-hop pedagogy. Buffington and Day (2018) focus on cipher (also called cypher in some articles), a term frequently mentioned in hip-hop historical literature (Lefebvre, 2012; Dattatreyan and Singh, 2020; Yang et al., 2022). Buffington and Day (2018) noted that “ciphers” is a central aspect of defining hip-hop pedagogy (p. 3).

According to the accepted definition of the cipher (Dodds, 2018; Levy et al., 2018; Crooke et al., 2021; Lee, 2022; Ozelkan, 2022), it can be explained as an action by those groups active in the hip-hop community using hip-hop elements to communicate and share ideas. On this basis, we briefly summarize three basic characteristics of the cipher as follows:

1. Cipher is dynamic. This simply means that the location where people conduct cipher changes with the ever-changing environment. For example, cipher can occur in many formal and informal learning environments, such as on campus, on the street (Lipenga, 2023), and online forums (Henze and Hall, 2018).
2. Cipher is shared. The core idea behind cipher is really seeing all the fantastic innovations that are happening (Li and Vexler, 2019). The idea is to encourage all those who conduct cipher to use creative elements of hip-hop, such as music and dance, to express and share their creativity (Langnes, 2018; Li and Vexler, 2019; Mabingo, 2022; Ozelkan, 2022). Of course, if every single one of us did not engage in cipher, cipher would be meaningless.
3. Cipher is learned. It is not biological; we do not inherit it. That means teachers need to master a range of approaches to help diverse students conduct cipher well. Foley (2016) clearly demonstrates how hip-hop arts teachers can equip young children with the critical skills needed for cipher. Given space limitations, an extended discussion of these perspectives would be impractical. Therefore, interested readers refer to other relevant sources (Fernando, 2018; Mangialardi, 2019; Broome and Munson, 2020; Kurt, 2020; Le Lay, 2020; Jenkins, 2021).

The article by Wheatley (2022) cited above as an example of hip-hop pedagogy supporting student learning demonstrates a commitment to catering to the shared nature of cipher. Similarly, Henze and Hall (2018) demonstrated the dynamic nature of cipher by discussing “how a New Literacies approach to hip-hop pedagogy gives students increased opportunities to foster dialogue related to race and power” (p. 265). For example, Henze and Hall (2018) noted that “students undergo rich literacy processes by dissecting multimedia hip-hop texts, ranging from YouTube videos to discussions on online fan forums” (p. 268). Here, YouTube videos and online fan forums reflect the diversity of cipher locations.

To sum up, then, as we can see, hip-hop is used to define hip-hop pedagogy by describing how hip-hop elements, hip-hop-related knowledge, and ciphers deliver content, enhances the existing learning environment, and enhances the interactions between the students or teachers. In addition, we found that the dynamic feature of the cipher allows students to learn at their own pace in formal or informal spaces.

Furthermore, within the papers included in this literature review, some of the key terms associated with hip-hop in its broadest sense are discussed more frequently than terms related to pedagogy, with hip-hop culture, breakdancing, graffiti, rap, and knowledge being the most frequently referenced. In fact, this uneven focus is clearly relevant to the lack of the concept of pedagogy itself in the definition of hip-hop pedagogy referenced in our introduction. Questions about the source of the imbalance-how it has come into existence, why it persists, and whether there are factors that discourage teacher educators from understanding the term pedagogy are beyond the scope of this review itself, but provide an important agenda for future research. The following section will present the template for defining hip-hop pedagogy.

### Possible explanations for the definition of hip-hop pedagogy

This section aims to present an updated, expanded definitions of hip-hop pedagogy that represents two key aspects of hip-hop pedagogy in context, namely the usage of the key element “hip-hop” and the concept of pedagogy itself. Before we present possible explanations for the definition of hip-hop pedagogy, we pause here to clarify the usage of the critical term hip-hop used to define hip-hop pedagogy and review definitions of pedagogy mentioned in the first section.

According to the previous two sections, we summarize the key term hip-hop is used to define the concept of hip-hop pedagogy in the following way:

1. Hip-hop elements (breakdancing, graffiti, DJs, MCs, and self-knowledge) are used to enhance student-teacher interaction (Adjapong and Emdin, 2015) or integrated into the teaching materials (Buffington and Day, 2018).
2. The dynamic feature of cipher is used to describe the teaching and learning environment, such as formal or informal learning spaces (Henze and Hall, 2018); the shared feature is usually expressed by verbs such as “supporting and celebrating” (Wheatley, 2022, p. 104), the learned feature means that cipher as an action, it needs to be acquired through learning (Foley, 2016).
3. Knowledge related to hip-hop, such as hip-hop history, hip-hop culture, hip-hop music, and hip-hop dance, are integrated into the teaching materials (Adjapong, 2021; Wheatley, 2022).

In addition, in the hip-hop pedagogy literature included in this article, the term pedagogy is usually explained as teaching approaches (Adjapong and Emdin, 2015; Adjapong, 2021; Wheatley, 2022), but many researchers already clearly argued that the understanding of pedagogy should not be limited to teaching approaches, rather pedagogy should focus on how the results of learning meet specific social and economic requirements (Best, 1988; Hinchliffe, 2000; Alexander, 2008; Shah and Campus, 2021). To this end, we present the following description of the definition of pedagogy:

1. Pedagogy can be defined as learning oriented toward social needs; learning aims to prepare students for the talent market and help them develop job-related skills while growing personally and intellectually.
2. As pedagogy, the results of learning must be measurable because the whole meaning of learning is to enable people to meet specific social, political, and economic requirements.

Based on these perspectives, we present the following possible options for defining hip-hop pedagogy. These examples clearly articulate all the critical terms for defining hip-hop pedagogy, namely the concepts of hip-hop and pedagogy.

1. Hip-hop pedagogy is defined as a learning experience through integrating hip-hop elements into teaching materials in formal or informal learning environments where students interact with instructors and other students and are not dependent on their physical location for participating in this learning experience. Learning aims to acquire the skills needed for specific social and economic development.

OR

2. Hip-hop pedagogy is a learning experience through integrating knowledge related to hip-hop into teaching materials in formal or informal learning environments where students engage with instructors and peers at a time of their convenience. The purpose of learning is to meet the skills needed in job markets.

OR

3. Hip-hop pedagogy is defined as learning activities through hip-hop elements in formal or informal learning environments. The dynamic feature of cipher allows students to choose whether to learn in a formal or informal learning space at their own pace. Students’ learning achievement will be measured by faculty utilizing rubrics to determine whether students acquire skills needed for specific social or economic development.

The objective and implication of proposing options with these critical terms are to ensure that the definitions which use these options will be complete in terms of the necessary information needed, such as the characteristics of the cipher, knowledge related to hip-hop, and the notion of pedagogy. This template will neither be bogged down in unnecessary details nor leave out any critical details.

## Conclusion

This article aims to identify the definition of pedagogy and determine the usage of the key element hip hop in the context of hip-hop pedagogy through a literature review. This choice has been made because most definitions of hip-hop pedagogy lack the concept of pedagogy. In other words, if the definition of pedagogy is not clarified, then future researchers, teachers, or students will probably use hip-hop pedagogy in ambiguous ways to spread misinformation. In this context, we define pedagogy as social goal-oriented learning based on the perspectives and key features of the included literature in this article. Of course, it is necessary to clarify that we hold out little hope for a grand consensus in academia because there are so many conceptual definitions and disagreements about what pedagogy is. Here, we also call for future researchers, teachers, and students in the hip-hop field should be as explicit as possible about what they are saying and what they are not saying, as pedagogy and education terminology can be easily confused and misinterpreted.

In addition, we propose an operational framework to standardize the definition of hip-hop pedagogy based on the perspectives and key features of the literature included in this article. This framework is designed to convey in the simplest terms what hip-hop pedagogy is for the purpose of informing educators and preparing them to use data. The limitation of this article is that it focuses only on definitions. Again, given space limitations, we did not expand the database search. Despite the limitations of this article, it is the first attempt to revise and update existing definitions of hip-hop pedagogy, and the definition will probably be widespread.

## Author contributions

ZY collected and organized the literature. MW reviewed the article content. XW discussed the article structure with other authors. All authors contributed to the article and approved the submitted version.

## Conflict of interest

XW was employed by company 'ShaanXi Branch of Guangzhou Bosha Architectural Design Institute'.

The remaining authors declare that the research was conducted in the absence of any commercial or financial relationships that could be construed as a potential conflict of interest.

## Publisher’s note

All claims expressed in this article are solely those of the authors and do not necessarily represent those of their affiliated organizations, or those of the publisher, the editors and the reviewers. Any product that may be evaluated in this article, or claim that may be made by its manufacturer, is not guaranteed or endorsed by the publisher.
